# ENGAGING OLDER ADULTS WITH ALZHEIMER’S DISEASE IN EXERCISE: IMPACT ON CAREGIVERS

**DOI:** 10.1093/geroni/igac059.2811

**Published:** 2022-12-20

**Authors:** Tai Sims, Kristine Talley, Joseph Gaugler, Cynthia Peden-McAlpine, Laura Kirk, Fang Yu

**Affiliations:** Minnesota State University, Mankato, Mankato, Minnesota, United States; University of Minnesota, Minneapolis, Minnesota, United States; University of Minnesota, Minneapolis, Minnesota, United States; University of Minnesota, Minneapolis, Minnesota, United States; University of Minnesota, Minneapolis, Minnesota, United States; Arizona State University, Phoenix, Arizona, United States

## Abstract

Informal caregivers provide the bulk of care for persons with Alzheimer’s dementia (PWAD) living at home, resulting in increased burden, and deteriorations in wellbeing and health. The stress process model hypothesizes characteristics of PWAD influence caregiver outcomes. Few studies have identified if engaging PWAD in exercise influences caregiver outcomes. This ancillary mixed methods study of a RCT evaluated the impact of a 6-month, moderate-intensity aerobic exercise intervention for community-dwelling PWAD on family caregiver burden, wellbeing and general health. Quantitative data was collected at baseline, 3, 6, 9 and 12 months using Zarit Burden Interview, Caregiver Strain Index, Caregiver Wellbeing Scale, and 36-item Short Form Health Survey. Qualitative data was collected at 6 and 9 months using semi-structured interviews. The convergent mixed methods design included ANCOVA analyses of quantitative and content analysis of qualitative data. Participants were 25 (17 intervention, 8 control) caregivers aged 34–86 years who were primarily white, females that lived with care-recipient. Based on group assignment, quantitative and qualitative results provided mixed insight on influence of intervention on burden and wellbeing, and found no influence on general health. Quantitative findings indicated caregiver characteristics of relationship with care-recipient, gender and education influenced wellbeing, and relationship with care-recipient, co-residence and education influenced general health; and caregiver characteristics did not influence burden. Qualitative findings suggest improved burden and wellbeing were consequences of respite and social support, rather than the exercise intervention. This study provides insight that integrating family caregiver components into community-based exercise programs may benefit PWAD and their family caregivers.

